# Odorant-odorant metabolic interaction, a novel actor in olfactory perception and behavioral responsiveness

**DOI:** 10.1038/s41598-017-10080-z

**Published:** 2017-08-31

**Authors:** Hassan-Ismail Hanser, Philippe Faure, Aline Robert-Hazotte, Yves Artur, Patricia Duchamp-Viret, Gérard Coureaud, Jean-Marie Heydel

**Affiliations:** 1 Centre des Sciences du Goût et de l’Alimentation, UMR 6265 CNRS/1324 INRA/Université de Bourgogne Franche-Comté, 9 boulevard Jeanne d’Arc, F-21000 Dijon, France; 2 Centre de Recherche en Neurosciences de Lyon, INSERM U1028/CNRS UMR 5292/Université Lyon 1, 50 avenue Tony Garnier, F-69007 Lyon, France

## Abstract

In the nasal olfactory epithelium, olfactory metabolic enzymes ensure odorant clearance from the olfactory receptor environment. This biotransformation of odorants into deactivated polar metabolites is critical to maintaining peripheral sensitivity and perception. Olfactory stimuli consist of complex mixtures of odorants, so binding interactions likely occur at the enzyme level and may impact odor processing. Here, we used the well-described model of mammary pheromone-induced sucking-related behavior in rabbit neonates. It allowed to demonstrate how the presence of different aldehydic odorants efficiently affects the olfactory metabolism of this pheromone (an aldehyde too: 2-methylbut-2-enal). Indeed, according to *in vitro* and *ex vivo* measures, this metabolic interaction enhances the pheromone availability in the epithelium. Furthermore, *in vivo* presentation of the mammary pheromone at subthreshold concentrations efficiently triggers behavioral responsiveness in neonates when the pheromone is in mixture with a metabolic challenger odorant. These findings reveal that the periphery of the olfactory system is the place of metabolic interaction between odorants that may lead, in the context of odor mixture processing, to pertinent signal detection and corresponding behavioral effect.

## Introduction

Olfaction is a sense of high biological value that is at the forefront of food intake, reproductive behavior and social behavior, and which is more globally involved in health and well-being in all animals throughout life, including in humans. The initial step of odor perception occurs in the nose. Here, odorant molecules interact with olfactory receptors (ORs) expressed by olfactory sensory neurons (OSNs) lying in the olfactory epithelium (OE). The rule is that one OSN expresses only one OR subtype^1–3^ in such a way that each OSN and its OR are considered as a single entity^4–6^. The number of olfactory stimuli is quasi-infinite, constantly changing and mainly represented by more or less complex mixtures of odorants. Therefore, despite the high number of genes encoding ORs, most OSN-OR entities interact with large sets of molecules^7^ and thus, by combining their broad receptive profiles, ensure the encoding of the olfactory world.

In OE, the “pure” olfactory neural events and odorant metabolization by olfactory metabolizing enzymes (OMEs)^8^ run in parallel. The OME superfamily includes various enzymes (cytochrome P450, carboxylesterases, glutathione-S-transferase, among others) that ensure successive biotransformation of odorants, leading to their degradation and deactivation into metabolites. Strikingly, an increasing body of literature (mainly in insects) has evidenced the critical function of OMEs in odorant clearance from the perireceptor environment, pinpointing that OMEs are essential to ensure optimal detection and processing of odorants at the level of the OSNs^9–12^. In adult mammals, there is evidence that some perturbations of OME activity directly affect the peripheral olfactory response (measured by electro-olfactograms^13^) and subsequent olfactory bulb signal integration^14^. Despite this, odorant metabolism events remain underestimated and not adequately taken into account, at any level from olfactory processing to consecutive behavior of the organism.

Facing a mixture of odorants, single OSNs reflect, throughout their electrophysiological responses, complex odorant-odorant interactions at the OR binding and intracellular signaling levels, which *in fine* result in agonistic and antagonistic effects at the OSN response level^15, 16^. Because OMEs have the same ligands as ORs, they have to deal with the limitless number of odorants. Thus, facing an odorant mixture, one may suppose that OMEs interact with a large set of components while being submitted to odorant-odorant interactions. On that basis, OME could differentially metabolize the mixture components, altering the availability of some odorants at the expense of others, which *de facto* may impact olfactory neural processing and perception.

In the present study, we tested this hypothesis from the molecular enzymatic level to the perception and behavioral levels in a well described animal model of odor-induced guided behavior, the newborn rabbit and its responsiveness to the mammary pheromone. Naturally contained in the rabbit milk effluvium, the mammary pheromone is a monomolecular signal, 2-methylbut-2-enal (2MB2) [the mammary pheromone being 2MB2, it will hereafter be termed 2MB2 for simplicity and clarity], involved in the interaction of rabbit neonates with the mother, their sucking behavior and early odor learning, and therefore their survival^17–21^. In the OE of newborn rabbits, it has been recently demonstrated that 2MB2 is metabolized by glutathione-S-transferase enzyme (GST), which belongs to the OME family, and that this metabolism is particularly active within the developmental time-window during which 2MB2 perception is vital for young rabbits (i.e., at birth compared to the weaning)^20, 22–24^; It is noteworthy that preferential substrates of GST are aldehydes, including 2MB2.

Here, by using *in vitro* and *ex vivo* approaches, we provide evidence that different aldehydes efficiently interact with 2MB2 at the level of OMEs, with the interaction resulting in an increase of 2MB2 olfactory availability. Moreover, we show that a similar increase occurs in the OE, i.e., in the nose of living rabbit pups. Such an increase is obtained by mixing 2MB2, used at a non-behaviorally efficient concentration, with a metabolic challenger: so blended, 2MB2 is perceived at concentrations under the “usual” perception threshold and triggers the sucking behavior in neonates. This perception is juxtaposed with the fact that electrophysiological OSN population responses are larger when stimulated with 2MB2 in mixture with the challenger odorant, than with 2MB2 alone.

More broadly, the present study demonstrates that odorant-odorant metabolic interactions between mixture components can differentially and quantitatively increase the availability of a given odorant. For that odorant, the interaction may then result in dramatic consequences on its perception and, therefore, on the behavioral response that it elicits. Moreover, the fact that the availability of 2MB2, a pheromone crucial for survival in rabbit neonates, is preserved here (i.e., less metabolized) at the expense of other close odorants, leads us to propose that metabolic events may constitute a lever for promoting the perception of some highly relevant biological signals. In other words, OME activity may be functionally tuned.

Altogether, our work provides original cues that help to bring a better understanding of the physiological mechanisms occurring during the peripheral olfactory process, which influence perceptual and behavioral responses.

## Results

### *In vitro* measures: selection and comparison of metabolic challengers of 2MB2

Aldehydes structurally related to 2MB2 and previously shown to be glutathione conjugated^23^ were screened to identify challengers for glutathione conjugation metabolism toward 2MB2. With this aim, OE homogenate of newborn rabbits was incubated for 80 min at 37 °C with glutathione and a binary mixture composed of 2MB2 and a potential challenger or a non-challenger of 2MB2. Analysis by HPLC-Corona Ultra RS of the supernatant containing glutathione-aldehyde conjugates was then performed in order to rate the ability of each tested aldehyde to affect the formation of glutathione 2MB2 conjugate. Enzymatic glutathione conjugations of 2MB2 were compared for 2MB2 alone and for 2MB2 + challenger mixtures at ratios of 1:1 or 1:3.

**Figure 1:**
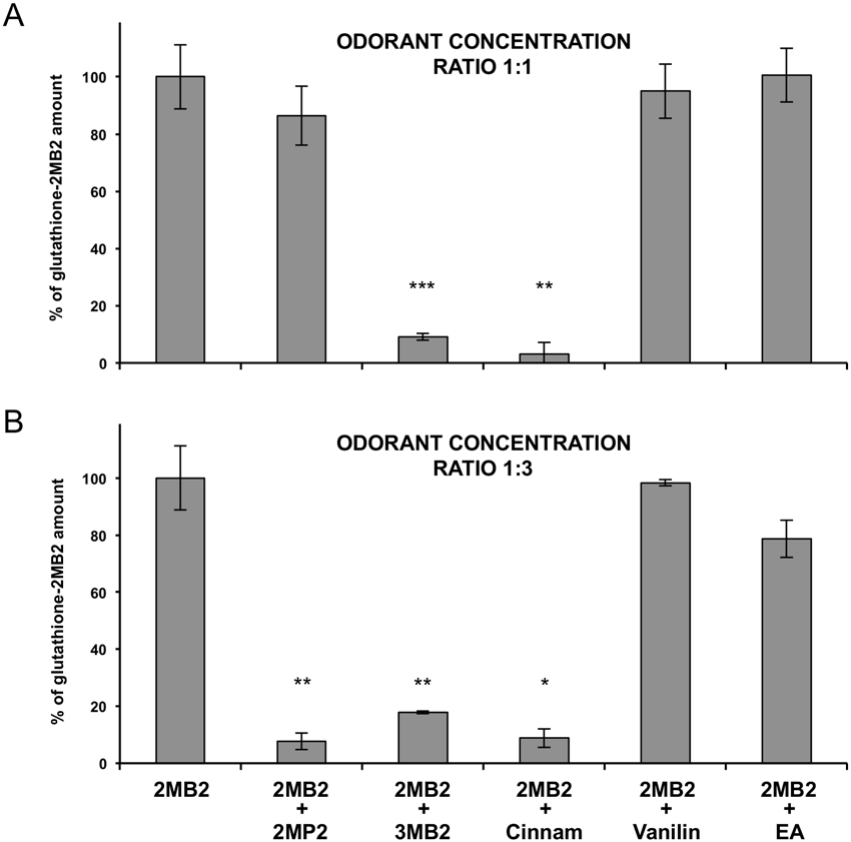
*In vitro* Glutathione conjugation of 2MB2 in presence of challengers. Glutathione conjugation of 2MB2 was determined by HPLC measurement, after incubation during 80 min at 37 °C of 2MB2 + reduced glutathione in presence of OE homogenate from newborn rabbits. 2MB2 enzymatic glutathione conjugation was compared to those obtained in presence of a challenger compound at equimolar concentration (ratio 1:1, **A**) and 3 times more concentrated (ratio 1:3, **B**). Results are expressed as % of the glutathione-2MB2 conjugate amount; 100% being obtained for 2MB2 alone. The % are means of n = 3–5 replicated measures ± SEM. *, ** and *** indicate significant differences (p ≤ 0.05, p ≤ 0.01 and p ≤ 0.001 respectively) between the control (2MB2 alone) and mixture conditions (Kruskal-Wallis multiple comparisons followed by a Conover-Iman *post-hoc* test). Odorant abbreviations: 2MB2: 2-methylbut-2-enal (the mammary pheromone), 2MP2: 2-methylpent-2-enal, Cinnam: Cinnamaldehyde, EA: ethyl acetate.

The *in vitro* analysis allowed the identification of three aldehydes that inhibited 2MB2 metabolism: 2MP2 (2-methylpent-2-enal), 3MB2 (3-methylbut-2-enal), and Cinnam (cinnamaldehyde) drastically reduced 2MB2 conjugation depending on the concentration ratio. At a 1:1 ratio, 3MB2 and Cinnam significantly lowered 2MB2 conjugation to 9.15% ± 1.2 (p = 0.0003) and 3.14% ± 3.95 (p = 0.006), respectively; at the same ratio, 2MP2 only weakly reduced 2MB2 conjugation, which remained at 86.4% ± 10.3 (Fig. 1A). At a 1:3 ratio, 2MP2, 3MB2 and Cinnam lowered 2MB2 conjugation to 7.7% ± 2.9 (p = 0.006), 17.9% ± 0.5 (p = 0.01) and 8.8% ± 3.2 (p = 0.031), respectively (Fig. 1B). By contrast, and as expected, vanillin and EA (ethyl acetate) did not significantly alter the production of glutathione-2MB2 conjugate (at 1:1 ratio: 95.0% ± 9.5 and 100.6% ± 10.3, at 1:3 ratio: 98.3% ± 1.1 and 78.7% ± 6.6, respectively; Fig. 1A,B). Vanillin and EA were used here as negative controls since they are metabolized by metabolic pathways other than glutathione conjugation^25, 26^. For subsequent experiments, the results led us to select 2MP2 and 3MB2 as 2MB2 conjugation challengers and EA as a non-challenger. Cinnam and the control vanillin were discarded because their partition coefficients were too weak according to what was needed in the subsequent *ex vivo* experiments.

### Ex vivo measures: kinetics of the inhibition of 2MB2 metabolism in presence of metabolic challengers

**Figure 2:**
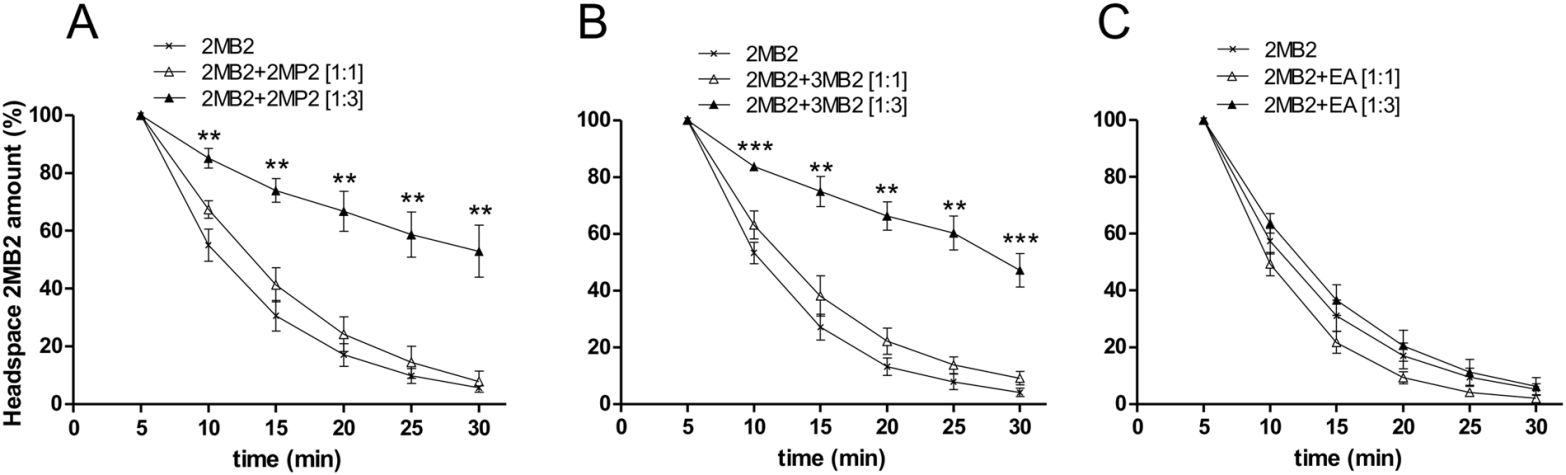
*Ex vivo* metabolism of gaseous 2MB2 in presence of challengers. Kinetic of disappearance of 2MB2 alone (cross), during the first 30 min, in presence of a total freshly collected newborn rabbit’s OE, compared to those obtained for 2MB2 mixed with 2MP2 (**A**), 3MB2 (**B**) or EA (**C**). Each mixture was tested at equimolar concentration (ratio 1:1; empty triangle) and with three times more concentrated challenger (ratio 1:3; solid triangle). The 2MB2 amount was determined, every 5 min by headspace gas chromatography measurement and 2MB2 peak integration analysis. Results plotted here are expressed as a percentage of 2MB2 in the headspace, relative to the initial amount 100% measured at 5 min for n = 3–8 replicates ± SEM. ** and *** indicate significant differences (p ≤ 0.01 and p ≤ 0.001 respectively) between the control (2MB2 alone) and binary mixtures conditions (Kruskal-Wallis multiple comparisons followed by a Conover-Iman *post-hoc* test). Same odorant abbreviations as in Fig. 1.

We previously developed a fast reliable and automated method to *ex vivo* measure endogenous global enzymatic odorant metabolism^23, 24^. Briefly, in this validated method, a fresh total explant of newborn rabbit OE was put in a sealed 20 ml vial, in which a known concentration of gaseous odorant was injected. Every 5 min, 250 µl from the headspace of the vial were automatically injected into a gaz-chromatograph for analysis of the remaining quantity of odorant. Appropriate controls validated the metabolism contribution of OE to the disappearance of the odorant from the headspace. This method, which preserves the cellular architecture and enzyme compartmentalization, was used here to evaluate the impact of odorant-odorant interactions on 2MB2 metabolism by GST. To assess the odorant-odorant metabolic interaction *ex vivo*, 2MP2 or 3MB2, identified as GST challengers from *in vitro* experiments, were here combined with 2MB2 at 1:1 and 1:3 ratios. The kinetics obtained with 2MB2 + 2MP2 at a 1:1 ratio demonstrated a rapid decrease of 2MB2, close to what we observed for 2MB2 alone: the residual 2MB2 in the headspace vials was 7.9% ± 3.7 compared with 5.7% ± 1.4 for 2MB2 alone (Fig. 2A). In contrast, for 2MB2 + 2MP2 at a 1:3 ratio, strong inhibition of 2MB2 metabolism was observed in the first 10 min (p = 0.003); 53.0% ± 9.0 of 2MB2 persisted at the end of the experiment (Fig. 2A). Similar results were obtained with the challenger 3MB2: no significant inhibition of the metabolism at a 1:1 ratio but significant inhibition at a 1:3 ratio from the first min (p = 0.001). The residual 2MB2 at the end of the procedure was 9.2% ± 2.4 for 2MB2 alone and 47.2% ± 5.8 for 2MB2 + 3MB2 (Fig. 2B). With the 2MB2 + EA control mixture, both 1:1 and 1:3 ratios showed kinetics close to that of 2MB2 alone, while similar residual 2MB2 amounts were found, 2.1% ± 0.4 and 6.2% ± 3.6, respectively (Fig. 2C).

### In vivo behavioral measures: consequences of metabolic interaction between 2MB2 and different aldehyde challengers

After demonstrating that interaction between 2MB2 and some close aldehydes at the OME level resulted in a decrease of 2MB2 metabolism, and thus an increase of its residual concentration, we investigated the impact of odorant-odorant metabolic interactions on rabbit neonate perception and behavior. In doing so, we focused on the typical sucking-related orocephalic response usually displayed by rabbit pups in response to efficient concentrations of 2MB2 (details are in Methods). Here, binary mixtures of 2MB2 (at 10^−9^ g/ml, i.e., a concentration level just below the perception threshold of 2.5 × 10^−9^ g/ml) and a challenger were presented to 2-day-old pups distributed in 3 groups. Each group differed according to the nature of the challenger: 2MB2 + 2MP2 (n = 15–36 pups from 3–7 litters/concentration), 2MB2 + 3MB2 (n = 20 pups from 4 litters) and 2MB2 + EA (n = 20–44 pups from 4–6 litters/concentration). In each group, the pups were tested for responsiveness to 2MB2 alone (at 10^−6^ and 10^−9^ g/ml, as a positive and negative control, respectively), to each of the challengers alone (at 10^−6^ g/ml) and to the binary mixture of 2MB2 + challenger at different concentrations of each odorant (see below).

**Figure 3:**
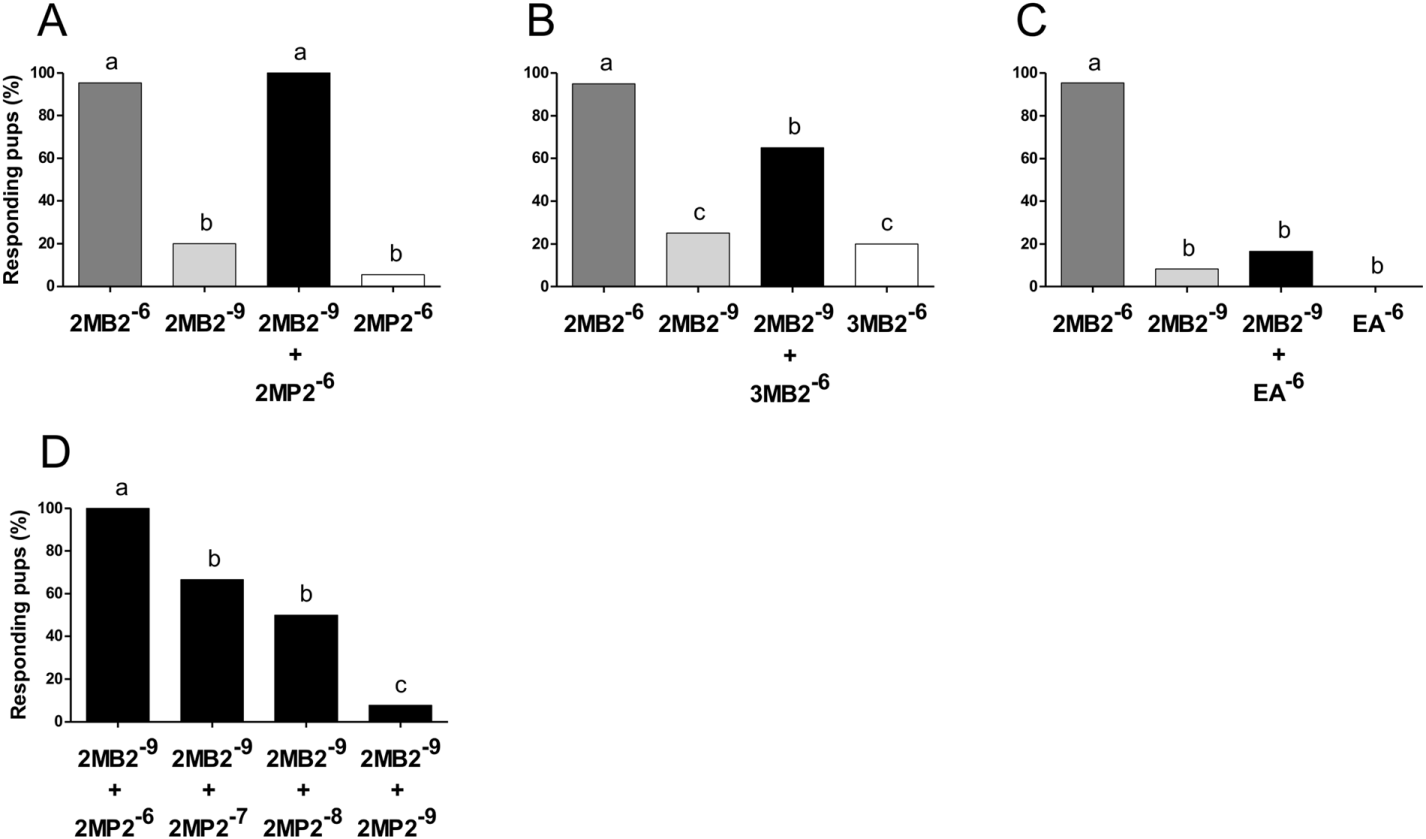
Behavioral responsiveness to 2MB2 alone or in binary mixture with 2MP2, 3MB2 or EA. Proportions of rabbit pups responding by orocephalic movements to different stimuli in the glass-rod test (n = 15 to 36 pups, from 3 to 7 litters depending on the groups). (**A–C)** Responsiveness to 2MB2 (the mammary pheromone) at 10^−6^ g/ml and 10^−9^ g/ml, illustrating positive and negative controls, compared to responsiveness to 2MP2 (**A**), 3MB2 (**B**) and EA (**C**) each singly presented at 10^-6^ g/ml, or at 10^−9^ g/ml in binary mixture with 2MB2. D: responsiveness to 2MB2 (10^−9^ g/ml) + 2MP2 (10^−6^ g/ml) mixture compared to three other mixtures containing the same concentration of 2MB2 (10^−9^ g/ml) and decreasing concentrations of 2MP2 (10^−7^ 10^−8^, 10^−9^ g/ml). Within each graph, distinct digits indicate statistical differences (p ≤ 0.05): χ² or Cochran test, for independent or dependent multiple comparisons, respectively, followed by a McNemar or χ² test for pairwise comparisons. Same odorant abbreviations as in Fig. 1.

As expected, in each group, almost all the pups (95.3%; range: 95–95.5%) responded to 2MB2 at 10^−6^ g/ml while only a few pups (17.8%; range: 8.3–25%) responded to 2MB2 at 10^−9^ g/ml (Fig. 3A–C). However, and strikingly, 100% of the pups displayed the typical orocephalic behavior in response to a mixture of 2MB2 10^−9^ + 2MP2 at 10^−6^ g/ml, i.e., as efficiently as to 2MB2 alone at 10^−6^ g/ml (p = 0.271; Fig. 3A). The reactogenic power of the mixture cannot be attributed to 2MP2 perception *per se*, since 2MP2 induced the orocephalic response in only 5.6% of the pups when presented alone at 10^−6^ g/ml (comparison with the mixture: p = 0.0001; Fig. 3A). Moreover, an additional control group of 23 pups (from 5 litters) tested with higher concentrations of 2MP2 displayed no response to this odorant (≤4.6% of responsiveness at 2MP2 10^−5^, 10^−4^ and 10^−3^ g/ml), whereas they strongly responded to 2MB2 at 10^−6^ g/ml (≥95.6%; p < 0.001 in all comparisons between 2MP2 and 2MB2) (see Supplementary Fig. S1). Similarly, when 2MB2 at 10^−9^ g/ml was mixed with another challenger, 3MB2 at 10^−6^ g/ml, pup responsiveness increased and reached 65% (p = 0.013), while it was only weak to 3MB2 alone (Fig. 3B). Conversely, in response to 2MB2 at 10^−9^ g/ml mixed with the non-challenger EA at 10^−6^ g/ml, pups were as weakly reactive to the mixture as to 2MB2 at 10^−9^ g/ml or EA at 10^−6^ g/ml singly delivered (p = 0.383 and p = 0.056 in the two comparisons; Fig. 3C).

Therefore, 2MP2 and 3MB2 appeared able to promote the pups’ behavioral responsiveness when binarily mixed with subthreshold concentrations of 2MB2. Moreover, our behavioral data support the notion that 2MP2 would be the most efficient metabolic challenger, compared to 3MB2 (comparison 2MB2 + 2MP2 *vs* 2MB2 + 3MB2: p = 0.0007). This is why we investigated the interaction of 2MP2 with 2MB2 at 10^−9^ g/ml in the next step, with 2MP2 being used at decreasing concentrations from 10^−6^ through 10^−7^, 10^−8^ and 10^−9^ g/ml (n = 24–26 pups from 6 litters/concentration). In doing so, the pups’ responsiveness to the binary mixture appeared to be concentration-dependent; the lower the concentration of 2MP2, the lower the responsiveness of the pups to the mixture (100%, 66.7%, 50% and 7.7%, respectively; general comparison: p = 0.0001; 2 × 2 comparisons between 2MB2 × 10^−9^: + 2MP2 × 10^−6^ *vs* each other mixture: p < 0.004; + 2MP2 × 10^−7^ or 2MP2 × 10^−8^ *vs* + 2MP2 × 10^−9^: p < 0.002; + 2MP2 × 10^−7^ *vs* + 2MP2 × 10^−8^: p > 0.05) (Fig. 3D).

### *Ex vivo* electrophysiological measures: peripheral responses to 2MB2 alone and in mixture with 2MP2 or EA

Here, we assessed whether the perception of subthreshold concentrations of 2MB2, when in mixture with a metabolic challenger, finds some origin in the peripheral olfactory processes of rabbit neonates. To do so, we recorded *ex vivo* populational responses of OSNs (electro-olfactograms, EOGs), from OE of newborn rabbits (n = 15, from 5 litters). The EOGs recordings aimed at analyzing the responses to 2MB2 alone or in the presence of 2MP2 (its best metabolic challenger) or EA (a non-metabolic challenger) at different concentrations and ratios. EOGs were simultaneously sampled from turbinates I to III; the latter did not show spatial differential sensitivity to the stimuli. Thus, the data were pulled and averaged for each preparation (hemi-head) and stimulus, regardless of the recording site. Overall, stimuli evoked robust EOGs ranking from 0.1 to 8.7 mV.

**Figure 4:**
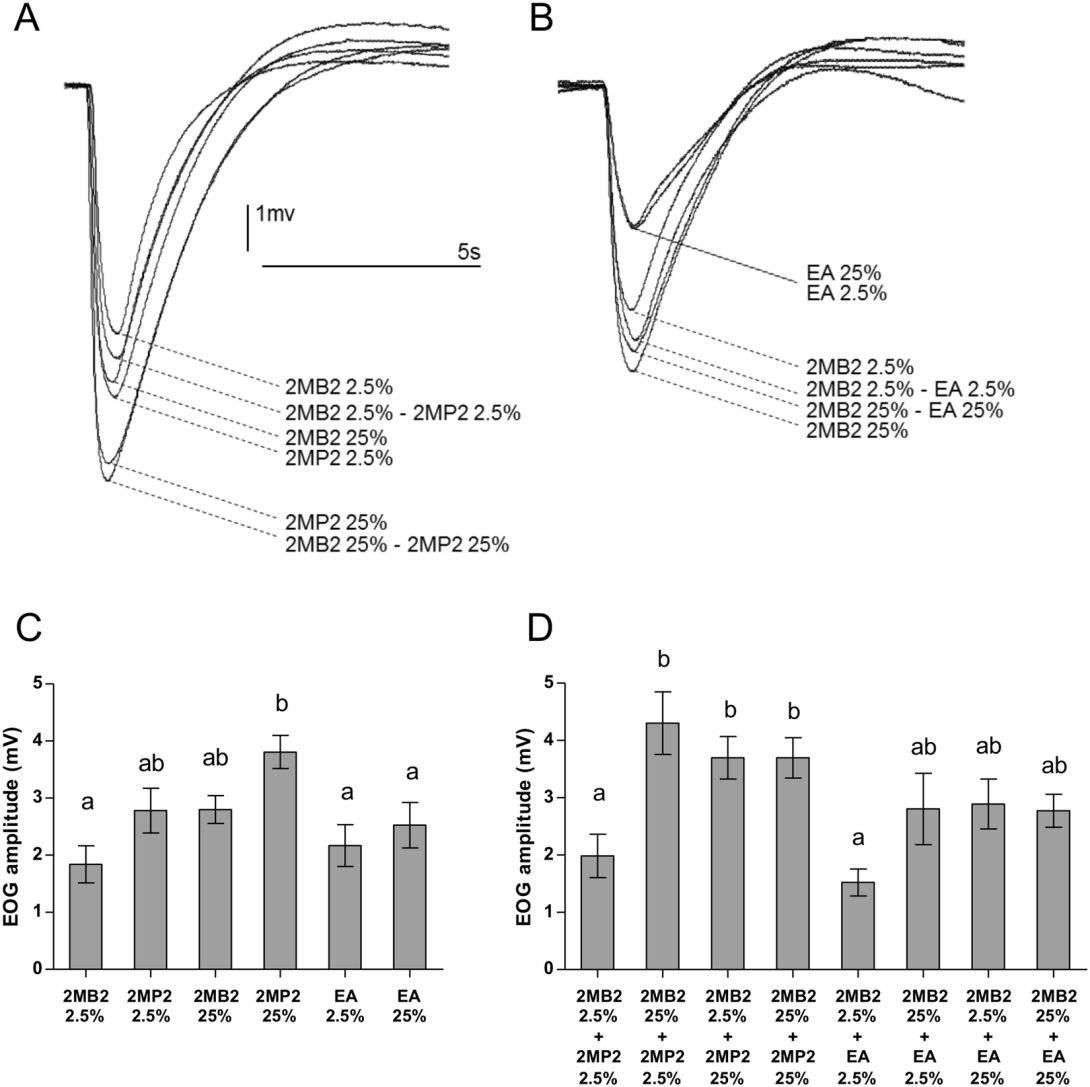
*Ex vivo* EOG responses to single odorants and binary mixtures including 2MB2. (**A**,**B**) Representative examples of EOGs (raw recordings). (**A**) EOGs recorded in response to 2MB2, 2MP2 and their binary mixtures at different concentrations (see Table 1). (**B**) EOGs recorded in response to 2MB2, EA and their binary mixtures at different concentrations (see Table 1). (**C**,**D**) EOG amplitudes are given as mean ± SEM averaged for the three recording sites and 3–5 repeated stimulations, for each stimulating condition. (**C**) Responses to single odorants; D: responses for binary mixtures. In each histogram, stimuli are sorted from the left to the right according to increasing calculated concentrations (see Table 1). Within each graph, distinct digits indicate statistical differences (p ≤ 0.05, Kruskal-Wallis multiple comparisons followed by a Conover-Iman *post-hoc* test). Same odorant abbreviations as in Fig. 1.

For 2MB2 and 2MP2 (singly or in mixtures), the superimposed recordings evidence that EOG amplitudes increase with the stimulus concentrations (Fig. 4A). By contrast, for EA and 2MB2 (singly or in mixtures), EOG amplitudes are not directly accountable to the stimulus concentrations (Fig. 4B, and see Table 1).

**Table 1:** EOG’s recording stimuli and their calculated concentrations in mol/l.

Stimulus	Calculated concentrations mol/l
2MB2 2.5%	1.17E-06
2MB2 25%	1.17E-05
2MP2 2.5%	1.01E-05
2MP2 25%	1.01E-04
EA 2.5%	1.32E-04
EA 25	1.32E-03
2MB2 2.5% + 2MP2 2.5%	1.13E-05
2MB2 25% + 2MP2 2.5%	2.18E-05
2MB2 2.5% + 2MP2 25%	1.02E-04
2MB2 25% + 2MP2 25%	1.13E-04
2MB2 2.5% + EA 2.5%	1.33E-04
2MB2 25% + EA 2.5%	1.44E-04
2MB2 2.5% + EA 25%	1.32E-03
2MB2 25% + EA 25%	1,33E-03

Single odorant and binary mixtures are ranked according to their increasing calculated concentration from the top-down.

The mean response values for single odorants at two concentrations (2.5 and 25% in vol/vol of saturated vapors; number of rabbit hemi-heads; n = 11–30, depending on the stimulus) are plotted in Fig. 4C: for 2MB2 and 2MP2, the graph shows that EOG amplitudes increased significantly with the concentrations (p = 0.001 and 0.04 respectively, pairwise comparison not shown, consistent with Fig. 4A). By contrast, EA, the most concentrated stimulus (Table 1), did not elicit the largest EOG and, furthermore, did not significantly increase as a function of its concentration (p = 0.3, pairwise comparison not shown, consistent with Fig. 4B). Overall, regarding single odorant, 2MB2, despite its hundred-fold lower saturated vapor pressure (SVP) concentration than EA, evoked EOGs of similar amplitude than the latter. Similarly, 2MP2, which is ten-fold lower than EA (SVP), displayed the highest amplitudes (especially 2MP2 25%), which are statistically different from those obtained for EA (p < 0.002). These results support that OE of newborn rabbits would be a propitious environment for 2MB2 and 2MP2 processing: the latter elicited larger responses at much lower concentrations than EA.

Focusing on the two aldehydes, 2MB2 and 2MP2, 2MP2 elicited the largest responses, as the most volatile and thus concentrated, in gaseous phase. Comparisons between the mixture EOG response values (Fig. 4D) showed that 2MB2 25% + 2MP2 2.5%, 2MB2 2.5% + 2MP2 25% and 2MB2 25% + 2MP2 25% elicited the highest responses: the latter are statistically different from those obtained for 2MB2 2.5% + 2MP2 2.5% and 2MB2 2.5% + EA 2.5% (p < 0.0003), but not from those obtained for 2MB2 25% + EA 2.5%, 2MB2 2.5% + EA 25% and 2MB2 25% + EA 25%.

As expected from some of our previous studies^16^, the mixture response amplitudes never consist in simple sums of those induced by their components. According to our conventions^16^, analysis of the interaction between mixture components requires to take as reference the response to the most efficacious component, here 2MP2 (or EA for the non challenger); a larger response to mixture characterizing a synergy interaction type. Thus, taking 2MP2 as a reference, pairwise tests applied on mixture responses showed that 2MP2 2.5% + 2MB2 25% induced responses significantly larger from 2MP2 2.5% (p = 0.013; interaction type: synergy) (see Supplementary Fig. S2, A–D). Because only 2MB2 or 2MB2 + 2MP2 are able to release the orocephalic behavior of rabbit neonates, we also looked at the comparison of 2MB2 response vs 2MB2 + 2MP2: it reveals that responses to 2MB2 + 2MP2 (2.5% + 25%) were significantly higher than responses to 2MB2 2.5% (p = 0.005; interaction type: synergy) (see Supplementary Fig. S2, E–H). In Figure S3 (available as Supplementary Figure) are illustrated the comparisons of EOG responses for mixtures including EA: whatever the direction of the comparisons (EA vs EA + 2MB2 or 2MB2 vs EA + 2MB2), no significant difference was found.

## Discussion

The OE environment faces a multiplicity of olfactory stimuli that are mainly mixtures of molecules, i.e., of odorants. A plausible intuitive consequence would be that all the molecular receptors in charge of interacting with olfactory stimuli should rather have broad spectral profiles, allowing them to interact with several of the odorants composing “odors”. Such broad spectra have been demonstrated for OSN-OR entities^7^ and olfactory binding proteins (OBP), which transport hydrophobic odorant molecules to ORs^27^. As regards ORs, their broad response spectra have a crucial functional consequence: mixtures of odorants give rise to odorant-odorant interactions, which govern the olfactory coding as expressed through synergic, suppressive or masking interactions^15, 16, 28^. By analogy, the present study assessed the hypothesis of occurrence of such interactions at the level of OMEs, and led us to show that they concretely exist and impact the neural and behavioral response.

*In vitro* analysis measurements of 2MB2-glutathione conjugates showed that 2MB2 metabolism was affected by the presence of some aldehydes in the mixture; notably, it can be strongly inhibited. The result that non-GST conjugated aldehydes, namely EA or vanillin, did not affect 2MB2 metabolism demonstrated that such an inhibition results from metabolic interaction toward GST. Our headspace gas-chromatography method for *ex vivo* measurement^24^ allowed us to select 2MP2 as the best challenger. Moreover, and most importantly, it led us to show that in the vial headspace, a high level of residual/available 2MB2 (no metabolized) resulted from 2MB2-2MP2 interaction.

From the behavioral point of view, 2MB2 usually triggers the typical sucking-related orocephalic response of newborn rabbits at concentrations ranging from 2.5 × 10^−9^ to 10^−4^ g/ml^29^. Strikingly, using the same paradigm here, 2MB2 + 2MP2 mixture promoted the behavior although 2MB2 was under the perception threshold concentration (10^−9^ g/ml) in the mixture. This infra-perception threshold may be permitted by the fact that more 2MB2 would remain non-degraded at the OME level when it is in mixture with 2MP2, at the expense of the challenger, as shown in our *ex vivo* headspace results. Thus, *in vivo* interactions between both odorants could increase 2MB2 availability in the vicinity of ORs, and would elicit larger OSN responses, the latter impacting the subsequent olfactory bulb processing^14^. *In fine*, the perception threshold would be reached for lower 2MB2 concentrations and the sucking behavior triggered even at those concentrations.

We previously proposed that the high level of 2MB2 metabolism in the rabbit neonate OE optimizes its clearance, in order to maintain the reactivity of OSNs to subsequent 2MB2 stimulation^23^. Actually, rabbit pups do not rapidly habituate to 2MB2, since after 4 or 5 min of exposure (which is the normal duration of a nursing episode in that species^17, 21, 30^) they often remain responsive to the pheromone (Coureaud, personal observation). The present results led us to go further and postulate that odorant-odorant interactions at the OME level could favor detection of a given stimulus –here 2MB2- depending on precise ecological requirements - here 2MB2 -, by decreasing its degradation to the benefit of qualitatively close components. At birth, the environment of rabbit pups may simultaneously expose them to 2MB2 and odorant challengers for GST conjugation. For instance, such challengers could be intrinsic constituents of rabbit milk (which includes more than 150 other volatile components in addition to 2MB2: among them, 21 - including 2MB2 - have been identified, at least 129 remain unidentified^18, 30^), of other maternal or siblings’ emissions or of the nest components. Regardless of the origin of odorant challengers, the present results led us to propose that the activity of OMEs could be finely tuned to ensure the clearance of aldehydes while enhancing the concentration of 2MB2 around the ORs. Thus, based on differential metabolism, such a precise balance would optimize the detection of the biological signal by increasing the signal-to-noise ratio, favoring the interaction with the mother and reinforcing the survival of pups. Indeed, rabbit pups are strongly dependent on successful milk intake to survive, especially during the first nursing episodes, which occur only once per day in this species^31–33^.

Our behavioral observations describing the perception of 2MB2 at subthreshold concentrations when it is mixed with a metabolic odorant challenger, paired with our headspace chromatography results, strongly support that the temporal dynamics of metabolic and neural sensory events are compatible. That ties back to our above-mentioned hypothesis that an increase of 2M2B availability in the vicinity of OSNs, due to the presence of a challenger, would directly alter the detection threshold of pups; here, “directly” refers to the fact that such a 2M2B increase would enhance the OSNs’ response to this relevant biological signal and thus trigger the neonatal behavior. Consistently, we have previously reported, in adult rats, a consequence of OME blockade on OSNs’ responses^13^: the same concentrations of single odorants evoked larger EOGs. In the same line, weakly metabolized (glucuronoconjugated) odorants in OE elicited higher electrophysiological responses than strongly metabolized odorants (recorded *in vivo* from olfactory bulb mitral cells)^34^ and the low expression of OME in germfree mice was correlated with higher EOGs to odorants, potentially attributable to a longer availability of the odorants in the vicinity of NSOs^35^. Here, the question is a bit more complex because we compared responses to single components *vs* mixtures. However, we observed that the peripheral neural olfactory processing of 2MB2 + 2MP2 mixtures results in larger EOG responses (up to +350%) than that of 2MP2 or 2MB2 alone. Under our conditions, 2MP2 2.5 + 2MB2 25% (taking 2MP2 as reference) and 2MB2 2.5 + 2MP2 25% (taking 2MB2 as reference) can be proposed as characterizing synergistic interactions between the aldehydes^15, 16^. However, since EOG responses are populational reponses and their increases in amplitude mirror both increase of individual OSN responses and recruited number of OSNs, only single OSN recordings would allow to go further and lead us to know if the synergistic interaction between the aldehydes is based on increase in 2MB2 availability, on co-stimulation on same OSNs, or both. In any event, the result that 2MP2 alone is never behaviorally reactive, whatever the concentration (even a high one), demonstrated that distinct OSN combinations ensure 2MB2 and 2MP2 encoding.

By exploring the involvement of metabolic events in olfactory peripheral processing, from the molecular enzymatic level to the behavioral output, we demonstrated for the first time that peripheral neural processing and metabolic events are intricately linked. Metabolic events emerge here as novel inescapable actors in olfactory peripheral encoding. The OSN coding ability for mixtures of odorants involves several successive and interacting complexity levels, which originate first in the ability of OR-OSNs to interact with large sets of molecules^7, 36^, second in binding interaction modes^28^, third in the plurality of intracellular signaling^37^ and fourth in ligand-induced selective signaling^38^. Here, we propose that metabolic events constitute a supplementary level of sophistication in peripheral odor processing. In the OE, odorant-odorant interactions would contemporaneously occur at the OR and OME levels. As a result, according to the physiological or ecological context, the metabolic events would differentially impact the concentrations of odorants, and thus odorant-odorant interactions at the OR level, which originate masking, suppression, or synergy mechanisms^15, 16, 28, 39, 40^, itself promoting the peripheral sensory output. As a consequence, the final stimulus perception and behavioral responsiveness would be altered.

The OSNs’ electrophysiological activity^41^, and more generally the olfactory function^42–44^, have been shown to be modulated by the internal metabolic state of animals (after postnatal day 2 in newborn rabbits responding to the mammary pheromone^42^). Indeed, circulating signals such as insulin, leptin or orexin and from other circulating hormones target the OE. Within the general physiological context - for instance, starvation in newborn rabbits responding to the mammary pheromone during the daily maternal visit - we propose that the peripheral olfactory metabolism would constitute an additional lever on which circulating hormonal signals can act. Altogether, the control exerted on olfactory cues by metabolic events could promote the pertinent detection of an odor signal and behavioral responsiveness to that signal, likely by ensuring the optimal balance between its concentration and clearance. In a more informed way, beyond these physiological considerations, understanding such mechanisms may help to exhaust the perception of certain odorants in fragrance mixtures for health, welfare and commercial purposes.

## Methods

### Animals

New-Zealand rabbits (Charles River strain, France) originated from the breeding colony of the Centre de Zootechnie (Université de Bourgogne, Dijon). Adult females and males were housed in individual cages and kept under a constant 12:12 h light: dark cycle (light on at 7:00 a.m.) with ambient air temperature maintained at 21–22 °C. Water and food (Lapin Elevage #110, Safe, France) were provided *ad libitum*. Two days before the expected day of parturition, a nest-box (0.39 × 0.25 × 0.32 m) was fixed to the cages of pregnant females. The day of delivery was considered postnatal day 0. To even out pup-female interaction, females’ access to the nest was allowed for 15 min per day at 11:30 a.m. (i.e., this procedure allowed mimicking the short daily nursing displayed by rabbit females^31^. We used 276 newborn rabbits on postnatal days 1 or 2, i.e. 30 pups (from 15 litters) for *in vitro* HPLC assays, 48 pups (from 10 litters) for *ex vivo* kinetic measurements of 2MB2 metabolism in presence of challengers, 183 pups (from 40 litters) for behavioral assays, and 15 pups (from 5 litters) for *ex vivo* electro-olfactogram (EOG) recordings. The local, institutional and national rules regarding the care and experimental uses of the animals were followed. Thus, all experiments were conducted in accordance with ethical rules enforced by French law, and were approved by the local Ethical Committee of the University of Burgundy (Comité d’Ethique de l’Expérimentation Animale Grand Campus Dijon; C2EA grand campus Dijon N° 105), and by the French Ministère de l’Education Nationale, de l’Enseignement Supérieur et de la Recherche (MNESR) under the no. 001273.01.

### Olfactory stimuli

The odorants were the mammary pheromone, i.e., 2-methylbut-2-enal (2MB2; CAS #497-03-0), and 3-methylbut-2-enal (3MB2; CAS #107-86-8), 2-methylpent-2-enal (2MP2, CAS #623-36-9), vanillin (CAS #121-33-5), cinnamaldehyde (Cinnam, CAS #104-55-2) and ethyl acetate (EA, CAS #141-78-6). These chemicals were purchased from Sigma-Aldrich (*Sigma-Aldrich*, Saint-Quentin Fallavier, France). For specific details about the concentration at which the odorants were used, please see the following sections.

### Collecting of OE samples

After animal decapitation, the OE was carefully dissected out from both the septum cartilage and turbinates, while being careful not to include respiratory epithelium in samples. Then, the samples were immediately placed into a sterile phosphate buffer saline (0.05 M, pH 7.4) and frozen in liquid nitrogen to be immediately stored at −80 °C for further experiments using tissue homogenate, or placed into a 21 ml headspace vial for extemporaneous headspace gas chromatography (GC) experiments.

### Preparation of OE homogenates

OE samples were homogenized in PBS using a Tissuelyser (Qiagen) for 2 × 1 min at 30 Hz. The tissue homogenates were centrifuged for 5 min at 4 °C and 12000 g. The supernatant homogenates corresponding to 8–10 animals were pooled and the protein content was measured by the technique of Lowry^45^ using bovine serum albumin as standard. Small aliquots of supernatant (200 µl) containing glutathione-transferases were stored at −20 °C.

### HPLC (Corona ultra RS Charged Aerosol Detector) assessment of enzymatic glutathione conjugation with 2MB2, in presence of potential metabolic challengers

Enzymatic incubations were carried out in a reaction mix containing 12 µg of protein/µl of tissues supernatant homogenates in PBS, 15 mM reduced glutathione dissolved in PBS and 300 mM 2MB2 alone or mixed with 300 mM (ratio 1:1) or 900 mM (ratio 1:3) of other chemicals: either putative metabolic challengers to 2MB2 (2MP2, 3MB2 and cinnamaldehyde) or non-challengers (EA and vanillin). The odorants were dissolved in absolute ethanol. The final incubation volume was 50 µl. After 80 min incubation at 37 °C, the reaction was stopped by adding 50 µl of a 25% CuSO_4_ solution and the incubations medium were centrifuged for 3 × 10 min at 4 °C and 13500 g. The supernatants containing glutathione-2MB2 conjugates, diluted at 1:3 in bidistillated water, were analyzed by a high-performance liquid chromatography method (HPLC).

The HPLC analyses were performed using Ultimate 3000 series system equipped with dual low pressure gradient pump with vacuum degasser, a thermostated autosampler (set to 15 °C), a thermostated column compartment (set to 30 °C) and Corona Ultra RS charged aerosol detector (Thermo Scientific Dionex, France). Nitrogen gas from nitrogen generator NM30LA (LGS, France), regulated at 35 psi, was introduced into the detector and the resultant gas flow rate was regulated automatically and monitored by the Corona ultra RS Charged Aerosol Detector device. Nebulizer temperature was set to 10 °C. Response range was set to 100 pA full scale. The reversed phase HPLC of glutathione-2MB2 conjugates analysis was performed on a Hypersil^®^ GOLD C18 analytical column (150 mm, 2.1 mm; 3 μm particle size; ThermoScientific, France) using a multistep gradient with (A) 0.1% TFA in MS grade water and (B) 0.1% TFA in MS grade methanol as mobile phase. Gradient elution began at 99.5% (A) and 0.5% (B). It was kept constant for 6 min, increased to reach 60% (A) and 40% (B) at 9 min, kept constant for 3 min and then reduced to reach 99.5% (A) and 0.5% (B) at 15 min during 5 min. The flow rate of the mobile phase was 0.6 ml/min during the 20 min analysis time and the injection volume was 5 µl of samples. Data processing was carried out with Chromeleon 7.2 software (Dionex, France) and peak area corresponding to glutathione-2MB2 conjugate was integrated. 2MB2 (and other aldehydes) react spontaneously (i.e., chemically) with glutathione in solution to form glutathione conjugate^23^. To determine the part of enzymatic glutathione conjugation of 2MB2, HPLC quantifications of the 2MB2 conjugates were systematically performed in presence (i.e., with endogenous enzymes) and absence (i.e., without endogenous enzymes) of homogenate, two conditions representing the total conjugation (enzymatic and non-enzymatic) and the non-enzymatic conjugation, respectively. Enzymatic glutathione conjugation was defined by subtracting the non-enzymatic conjugation part from total conjugation. Final results are expressed as a percentage of the glutathione-2MB2 conjugate amount (formed in binary mixture with another odorant); the 100% reference being the amount of glutathione-2MB2 conjugate obtained for 2MB2 alone.

### Gas-liquid partition coefficient of the odorants (2MB2, 3MB2, 2MP2 and EA)

The aim of injecting a known gaseous odorant amount in the headspace above the olfactory mucosa made necessary the determination of the odorant gas-liquid partition coefficient. The latter was realized by using the Phase Ratio Variation (PRV) method^46^, as previously described by automatic equilibrium headspace - gas chromatography^24^. Briefly, aqueous solution of the 2MB2 or EA was prepared with the analyte concentration of 20 ppm and 100 ppm respectively. Then, increasing volumes (0.1, 0.2, 0.3, 0.5, 0.75, 1, 2 and 5 ml) of each solution were placed into 21 ml headspace vials and sealed with magnetic caps (Chromoptic, France). Each vial was then incubated under stirring at 37 °C overnight (allowing equilibrium between gas and aqueous phase) and 1 ml of sample headspace was injected by the automated Gerstel Multipurpose Sampler (MPS2) into a GC (Agilent 7890 A, Agilent Technologies) for measuring the area corresponding to the analyte peak. Then, by means of regression analysis of plotted area peak results, we calculated a partition coefficient value of 0.0065 ± 1.13 × 10^−5^ for 2MB2 and 0.0125 ± 2.26 × 10^−3^ for EA. Surprisingly, the molecular peculiar characteristic of 3MB2 and 2MP2 made the PRV method unusable to determinate the gas-liquid partition coefficient for these compounds. Alternatively, these two analytes were dissolved in a constant volume of bidistillated water (10 ml) in order to obtain, for each, the concentrations of 50 mg/l, 100 mg/l, 500 mg/and 1000 mg/l). These preparations were placed into headspace sealed vials and incubated overnight under stirring at 37 °C, before injection of the headspace sample (1 ml) into the GC. Concurrently, a calibration curve was realized by liquid injection of the analytes (1 µl in split/splitless mode, SSL) in dichloromethane at the following increasing concentrations: 0, 1, 5, 10, 25, 50, 100, 250, 500 and 1000 mg/l. The calibration curve was used to determinate the amount of analytes in the headspace samples and the partition coefficient values of 0.00197 ± 2.41 × 10^−4^ for 3MB2 and 0.0099 ± 1.01 × 10^−3^ for 2MP2 at 37 °C were calculated. Partition coefficients allowed then to calculate, for hermetic contents, the liquid concentration necessary to obtain, after equilibrium, the desired gaseous concentrations of odorants.

### Headspace gas chromatography instrumentation

The measurements were made on a gas chromatograph Agilent 7890 A (Agilent Technologies, Santa Clara, CA) coupled with flame ionization detector (GC-FID). A DB-WAX capillary column (30 m × 0.32 mm, film thickness 0.5 µm, J&W Scientific, Folsom, CA, USA) was used. Samples injections were made using a 1 ml gas-tight syringe with the automated Gerstel Multipurpose Sampler MPS2 into an injector split/splitless. The volume of the headspace vial was 21 ml. The optimum conditions for gas chromatography were as follows: two oven temperature was used to ensure best resolution peaks and non-overlapping elution times of compounds: 120 °C isothermic for 2MB2 and EA analysis and 100 °C isothermic for 3MB2 and 2MP2 analysis; the velocity of helium was 42 cm.s^−1^; detector and injector temperatures were 250 °C and 240 °C, respectively. For the FID detector, air and H_2_ flow rates were 400 ml/min and 30 ml/min, respectively. Carrier injector used a 5:1 split ratio.

### *Ex vivo* measurements of 2MB2 metabolism kinetic by headspace gas chromatography in presence of potential metabolic challengers

The 2MB2 metabolism kinetic has been previously described and validated with this headspace gas chromatography method^24^. Here, the 2MB2 kinetic of metabolization was compared to those observed in presence of another odorant, i.e., a putative metabolic challenger or not. The binary mixtures were made at different ratios. For experiments using 1:1 ratio, a 10 ml volume of a 3870 mg/l 2MB2 solution was placed into a headspace vial (volume: 21 ml) with 2120 mg/l 2MP2 or 10660 mg/l 3MB2 or 1680 mg/l EA solution, and stirred at 180 rpm overnight at 37 °C to obtain a 21 mg/l gaseous concentration of odorant in the headspace vial. For experiments with the 1:3 ratio, a 10 ml volume of a 3870 mg/l 2MB2 solution was placed into a headspace vial (volume: 21 ml) with 6360 mg/l 2MP2 or 31980 mg/l 3MB2 or 5040 mg/l EA solution, and stirred at 180 rpm overnight at 37 °C to obtain a 21 ppm gaseous concentration of 2MB2 and 63 ppm gaseous concentration of challengers or non-challengers in the headspace vial. One milliliter of the gas fraction from the headspace vial was introduced by the MPS2 autosampler into another vial containing a freshly dissected explant of OE (destination vial penetration was 45 mm with 100 µl/s add speed). Concentrations of the odorants in the gas phase above the explant of OE are thus of 1 ppm for 2MB2 and 1 or 3 ppm for challengers or non-challengers. Then, every 5 min during 30 min, 250 µl sampled from the headspace fraction of this vial containing odorant and OE was injected into the GC. The obtained kinetic curve representing the area of 2MB2 peaks was plotted and computed. Thus, for the different experimental conditions, i.e., 2MB2 alone and 2MB2 with a challenger or with a non-challenger odorant, the remaining (not metabolized) 2MB2 amount in the headspace vial was measured in percent and, compared to the reference 100% corresponding to the 2MB2 amount present at the first measurement by the GC (time 5 min).

### Behavioral assays

A 10-sec presentation of a glass rod (20 cm long, 0.4 cm in diameter), carrying one of the stimuli right under the pup nares, was used, as validated in previous studies^17, 18, 20, 22, 29, 47^. A stimulus was considered as behaviorally active when it released in pups the typical and not ambiguous head-searching movements (vigorous, low amplitude horizontal and vertical scanning movements displayed after stretching movements towards the rod) usually followed by grasping movements (labial seizing of the rod extremity) that newborn rabbits normally displayed in contact with the mother during nursing. Conversely, a stimulus was considered as inactive when it elicited no other response than sniffing. To minimize litter effects, each main experimental group was drawn from four to seven litters, with a maximum of five pups tested per litter in a given group (n = 15–36 pups/experiment). Each pup participated in only one experiment but was successively tested with 2–4 stimuli: 2MB2 alone systematically, and 3MB2, 2MP2 and/or EA, singly or in binary mixture with 2MB2 (an additional control group of 23 pups from 5 litters was tested to 3 concentration steps of 2MP2 and one of 2MB2). 2MB2 alone was used at two concentrations: 10^−6^ g/ml, which is maximally active on neonatal orocephalic behavior, and 10^−9^ g/ml, which is just below the perception threshold of the molecule^29^. The other single odorants were all used at 10^−6^ g/ml (a concentration at which they were fully perceptible), except 2MP2 which was also used at 10^−5^, 10^−4^ and 10^−3^ g/ml for control reasons in the additional group. The binary mixtures were composed of 2MB2 at 10^−9^ g/ml and another odorant at 10^−6^, 10^−7^, 10^−8^ or 10^−9^ g/ml, depending on the experiments. All stimuli were diluted in distilled water. The successive stimuli were presented at intervals of 120 s. The order of stimulus presentation was systematically changed from one pup to another coming from the same litter. If a pup responded to a stimulus, its muzzle was softly dried before the next stimulation. The pups were immediately reintroduced to the nest after testing.

### *Ex vivo* EOG recording

For electrophysiological recordings, the rabbit neonate dissection was the same as previously described for rats^41^. Briefly, each pup was anesthetized with equitesine (a mixture of pentobarbital and chloral hydrate; i.p. injection of 0.4 ml/100 g). Animals were then quickly decapitated and the head was halved. In each half-head, the septal wall, lined with the septal mucosa, was gently removed to uncover the nasal turbinates and give access to turbinates I, II and III for recordings (numbered from front to rear). Recordings were carried out on turbinates because, first, the latter longer resist to drying than septal mucosa and second, robust responses to 2MB2 have previously been described here in newborn rabbits^48^. The preparation was placed within water moist cotton in order to maintain a moistened environment during each entire recording session; in these conditions, turbinates kept a good aspect and reactivity upon several hours. EOG recordings were performed simultaneously in the three zones using homemade silver wire electrodes ended with a silver ball plated with AgCl. The electrodes diameter was about 500–700 µm. Electrodes were gently placed at the surface of the mucosa using micromanipulators. Recording visualization and acquisition was made on line using homemade Neurolabscope© software. The EOG signals were amplified by three conventional amplifiers (WPI, DC 30 Hz cut-off). The recordings were analyzed off line with Clampfit© (Axon Instrument software).

The odorants used for EOG recordings were 2MB2, 2MP2 and EA. Single odorants were available at 2.5 or 25% of the saturated SVP concentration which are: 4.68 × 10^−5^, 4.03 × 10^−4^ and 5.29 × 10^−3^ mol/l for 2MB2, 2MP2 and EA, respectively. The olfactory stimuli were delivered in gas phase throughout an olfactometer derived from a previous description^49^. They consisted in 0.3 sec square pulse at 80 ml/sec flow rate. The odorant stimulations were interspaced at least by 60 sec. The three odorants were delivered singly or in binary mixtures consisting in 2MB2 + 2MP2 or 2MB2 + EA. For each stimulus, the concentrations 2.5% and 25% (in proportion of SVP) could be combined in order to obtain four ratio and concentration combinations for each mixture. Thus, the final set of stimuli, representing a total of 14 conditions of stimulation, is given in Table 1. The stimulation and recording paradigm included 3–5 successive repetitions of each of the 14 stimulations; the inter-stimulation time intervals was 60 sec till 90 sec for the highest concentrations.

### Statistics

Glutathione conjugation analysis: 2MB2 conjugation in presence of other tested odorants was compared to the control condition of 2MB2 conjugation without other odorant. For a given ratio (1:1 or 1:3), Kruskal-Wallis multiple comparisons were done with determination of the p-value by Monte Carlo procedure. If the Kruskal-Wallis test demonstrated a significant difference, a Conover-Iman *post-hoc* test was performed between the control condition and each binary mixture to determine differences. For *ex vivo* metabolism measurement, the previous procedure was performed for each point of the kinetic, to compare the remaining amount of 2MB2 in mixture to the remaining amount of 2MB2 alone. For behavioral assays, the frequencies of responding pups were compared using the χ² test of Pearson when the groups were independent (i.e., distinct groups tested for their response to a same stimulus) or the Cochran’s Q test when the groups were dependent (i.e., pups from a same group tested for their response to distinct stimuli). When the Cochran’s Q or χ² tests were significant, McNemar or χ² tests were done respectively for pairwise comparisons. For *ex vivo* EOG recordings, multiple comparisons of EOG amplitudes were performed by Kruskal-Wallis test followed, if significant, by a Conover-Iman *post-hoc* test between each stimulus considered. Wilcoxon tests were performed for dependent pairwise comparisons on EOG responses obtained in same hemi-heads between 2MB2 alone and 2MB2 in mixture, for a given concentration of 2MB2. In all analysis, statistical significance was determined with two-tailed tests, an asymptotic determination of p-value (except for 2MB2 analysis of conjugation and metabolism), and a p-value threshold of 0.05.

## Electronic supplementary material

Supplementary Dataset
