# Supplementary material for: Odorant-odorant metabolic interaction, a novel actor in olfactory perception and behavioral responsiveness
Source: Sci Rep. 2017 Aug 31;7:10219. doi: 10.1038/s41598-017-10080-z (PMC5579276; doi:10.1038/s41598-017-10080-z)
Supplement: Supplementary file 1 — Supplementary Dataset [file 41598_2017_10080_MOESM1_ESM.doc]

Supplementary Information

**Odorant-odorant metabolic interaction, a novel actor in olfactory perception and behavioral responsiveness**

Hassan-Ismail Hanser^1^, Philippe Faure^1^, Aline Robert-Hazotte^1^, Yves Artur^1^, Patricia Duchamp-Viret^2^, Gérard Coureaud^2*^ & Jean-Marie Heydel^1*^

^1^Centre des Sciences du Goût et de l’Alimentation, UMR 6265 CNRS / 1324 INRA / Université de Bourgogne Franche-Comté, 9 boulevard Jeanne d’Arc, F-21000 Dijon, France. ^2^ Centre de Recherche en Neurosciences de Lyon, INSERM U1028 / CNRS UMR 5292 / Université Lyon 1, 50 avenue Tony Garnier, F-69007, Lyon, France.

*Correspondence and requests for materials should be addressed to J.M.H. (email: jean-marie.heydel@u-bourgogne.fr) or to G.C. (email: gerard.coureaud@cnrs.fr).

**Figure S1. Behavioral responsiveness to high concentrations of 2MP2.**

Proportions of rabbit pups (n=23, from 5 litters) responding by orocephalic movements in the glass-rod test to 2MB2 (the mammary pheromone) at 10^-6^ g/ml as a positive control, and to 2MP2 at 10^-5^, 10^-4^ and 10^-3^ g/ml. Distinct digits indicate statistical differences (p ≤0.001): Cochran test for dependent multiple comparisons, followed by McNemar test for pairwise comparisons. Same odorant abbreviations as in Figure 1.

**Figure S2. Vector representations of EOG amplitude variations: comparison between single component and mixture, for 2MB2 and 2MP2.**

A, B, C, D: reference compound 2MP2 at 2.5 or 25%. E, F, G and H: reference compound 2MB2 at 2.5 or 25%. In each graph, the vectors (full lines) correspond to the measures gathered from each half-head (the measures obtained from the three recording sites and repeated stimulations were averaged for each stimulating condition). Each vector illustrates the EOG amplitude variations (in %) between the responses to the reference component and mixture. The mean variation is given by dotted lines. * and ** indicate significant differences (p≤0.05 and p≤0.01 respectively, Wilcoxon paired analyses). Same odorant abbreviations as in Figure 1.

**Figure S3. Vector representations of EOG amplitude variations: comparison between single component and mixture, for 2MB2 and EA.**

A, B, C, D: reference compound 2MB2 at 2.5 or 25%. E, F, G and H: reference compound EA at 2.5 or 25%. In each graph, the vectors (full lines) correspond to the measures gathered from each half-head (the measures obtained from the three recording sites and repeated stimulations were averaged for each stimulating condition). Each vector illustrates the EOG amplitude variations (in %) between the responses to the reference component and mixture. The mean variation is given by dotted lines. No significant differences (Wilcoxon paired analyses). Same odorant abbreviations as in Figure 1.
